# Supplementary figures and images for: Chronic adolescent exposure to cannabis in mice leads to sex-biased changes in gene expression networks across brain regions
Source: Neuropsychopharmacology. 2022 Aug 22;47(12):2071–80. doi: 10.1038/s41386-022-01413-2 (PMC9556757; doi:10.1038/s41386-022-01413-2)

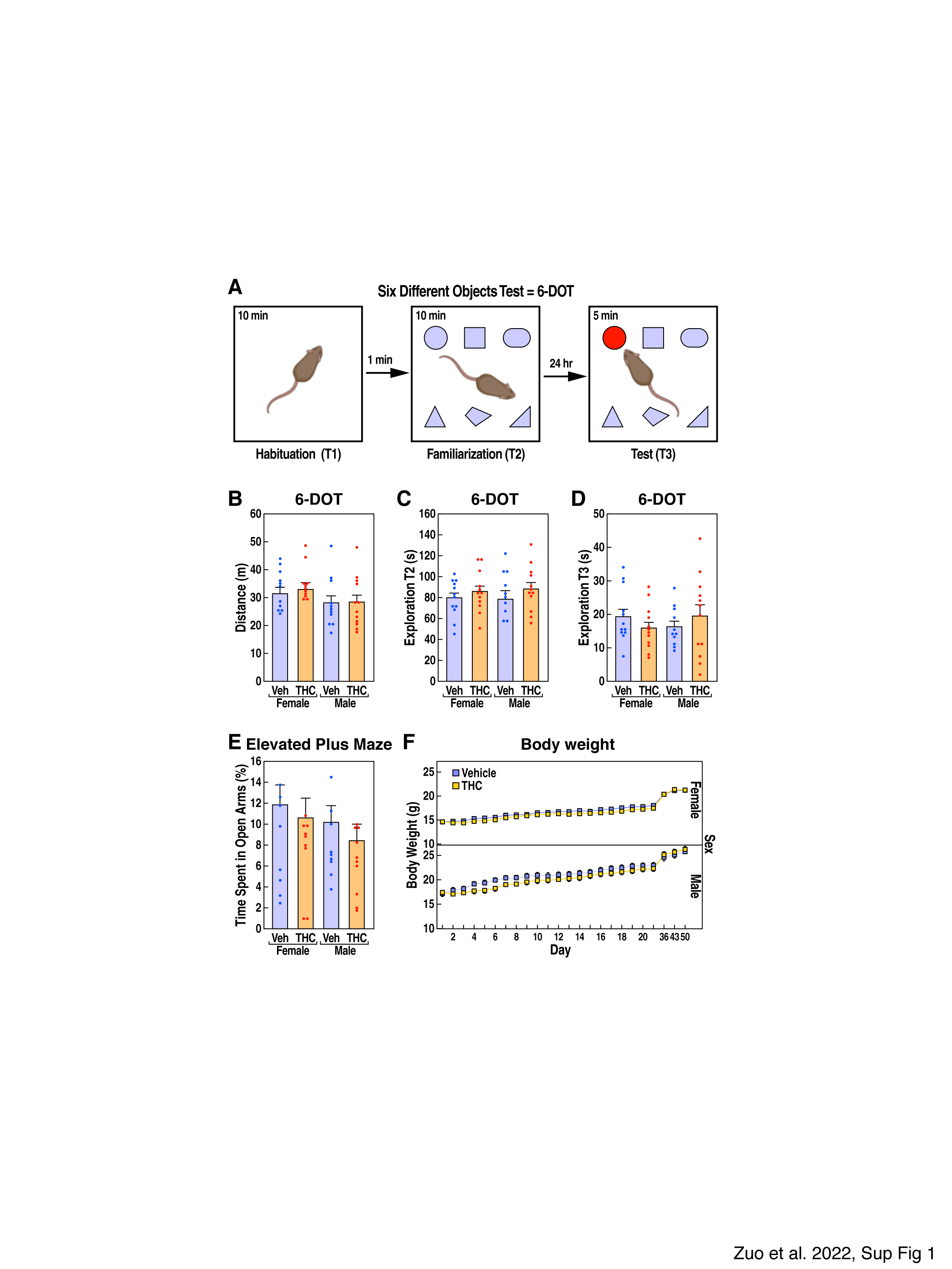

Supplement: Supplementary file 8 — Supplementary figures [file 41386_2022_1413_MOESM8_ESM.zip › Zuo et al. 2022, Sup Fig 1.tif]

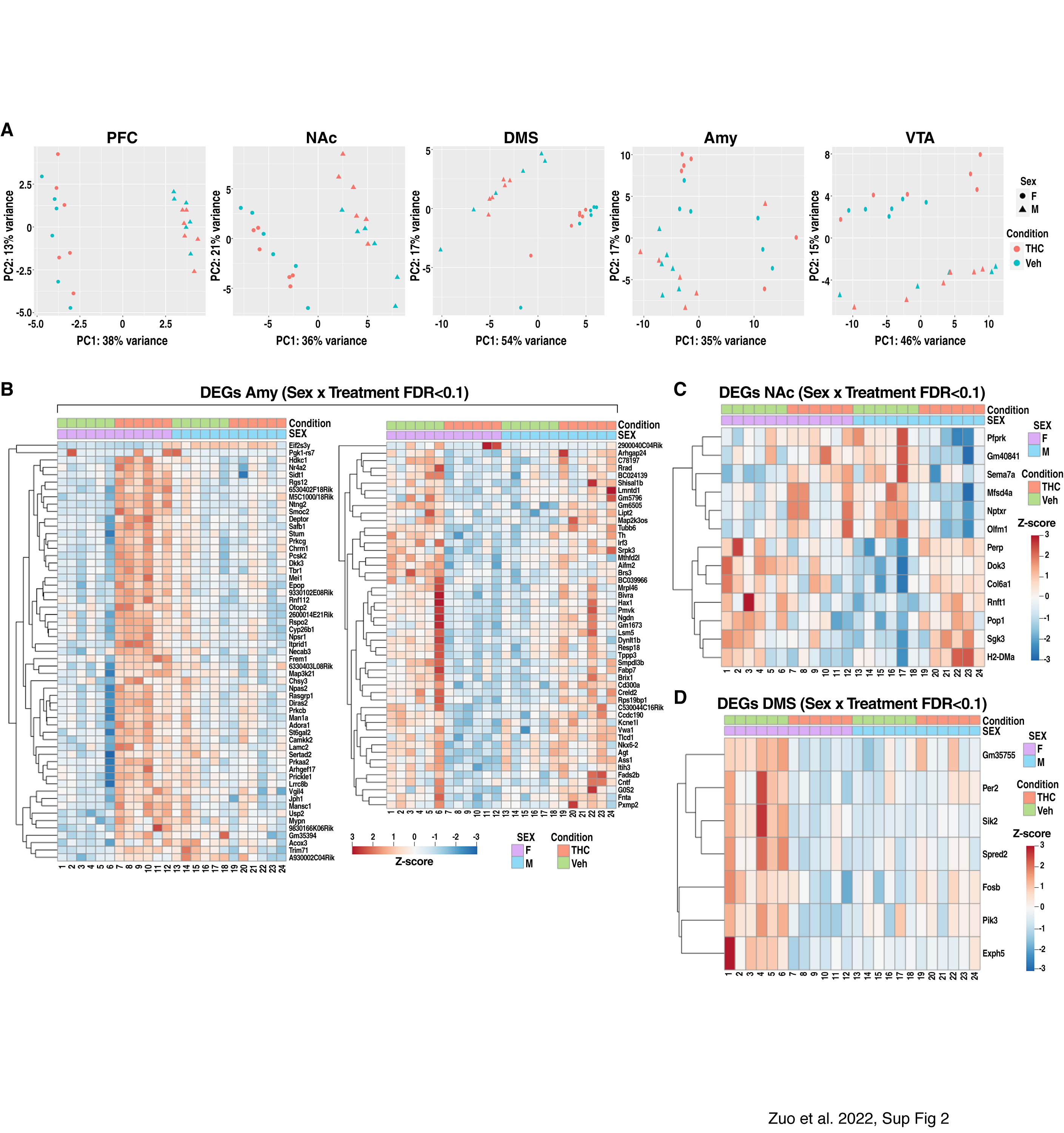

Supplement: Supplementary file 8 — Supplementary figures [file 41386_2022_1413_MOESM8_ESM.zip › Zuo et al. 2022, Sup Fig 2.tif]

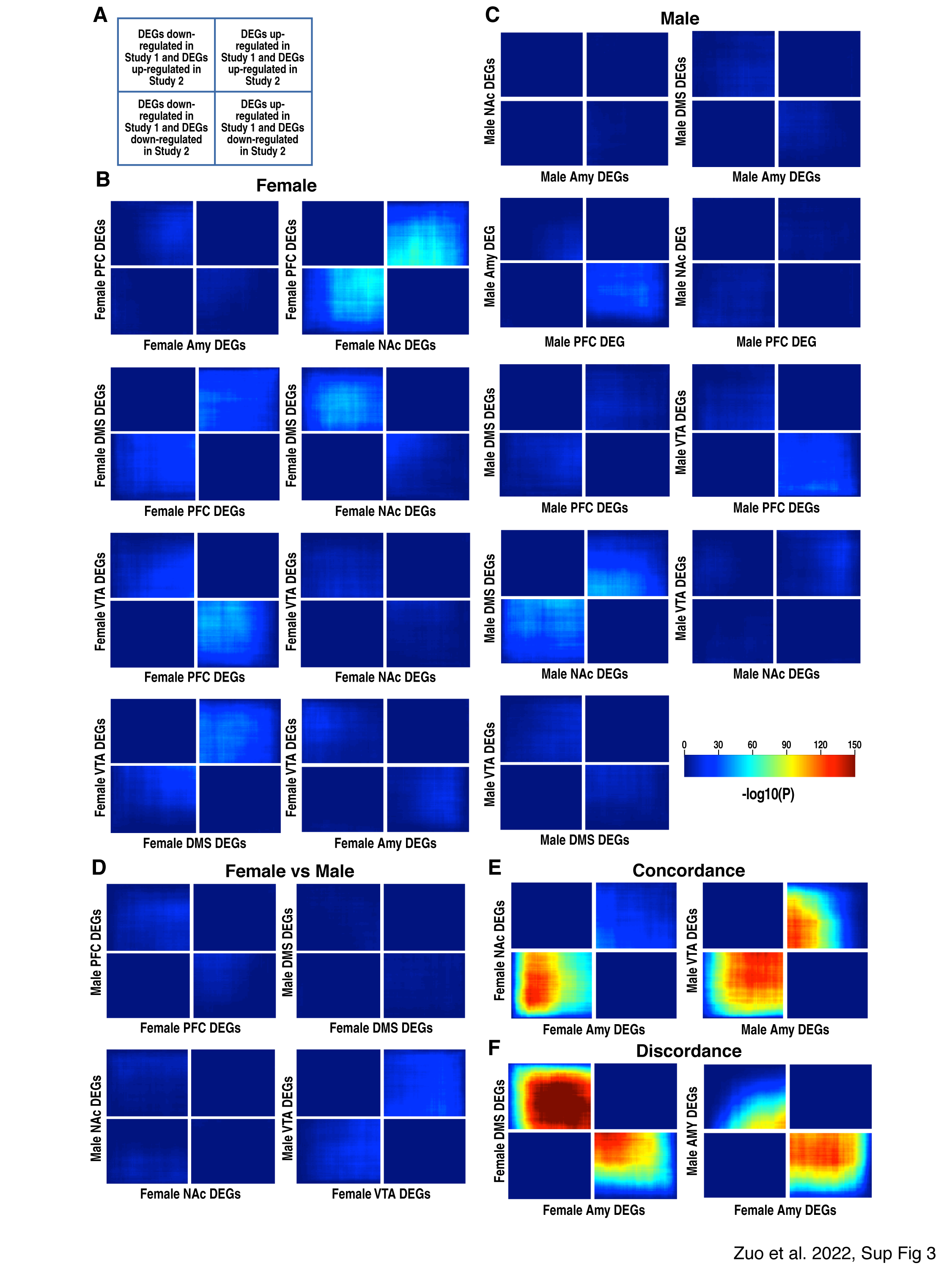

Supplement: Supplementary file 8 — Supplementary figures [file 41386_2022_1413_MOESM8_ESM.zip › Zuo et al. 2022, Sup Fig 3.tif]

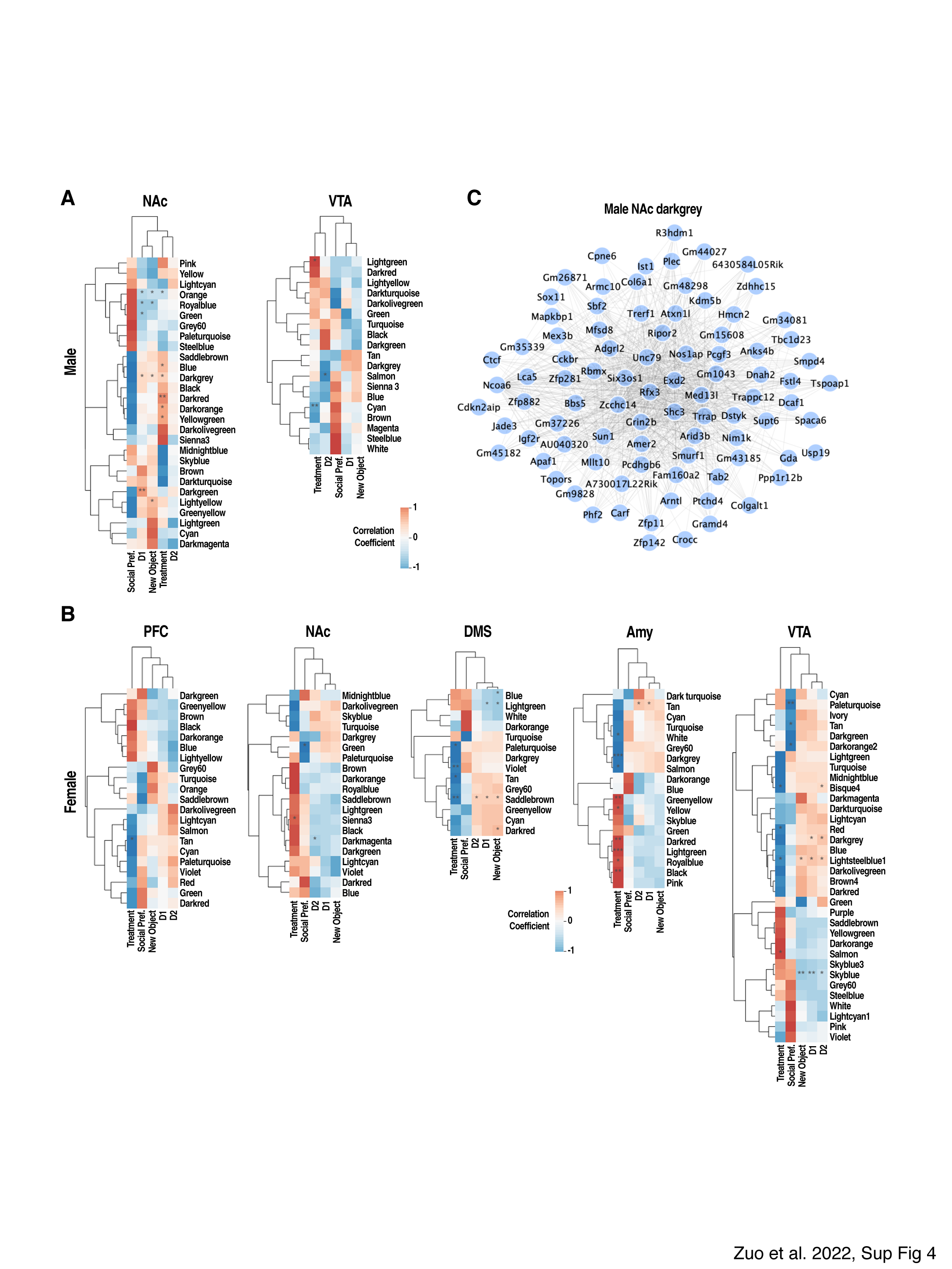

Supplement: Supplementary file 8 — Supplementary figures [file 41386_2022_1413_MOESM8_ESM.zip › Zuo et al. 2022, Sup Fig 4.tif]

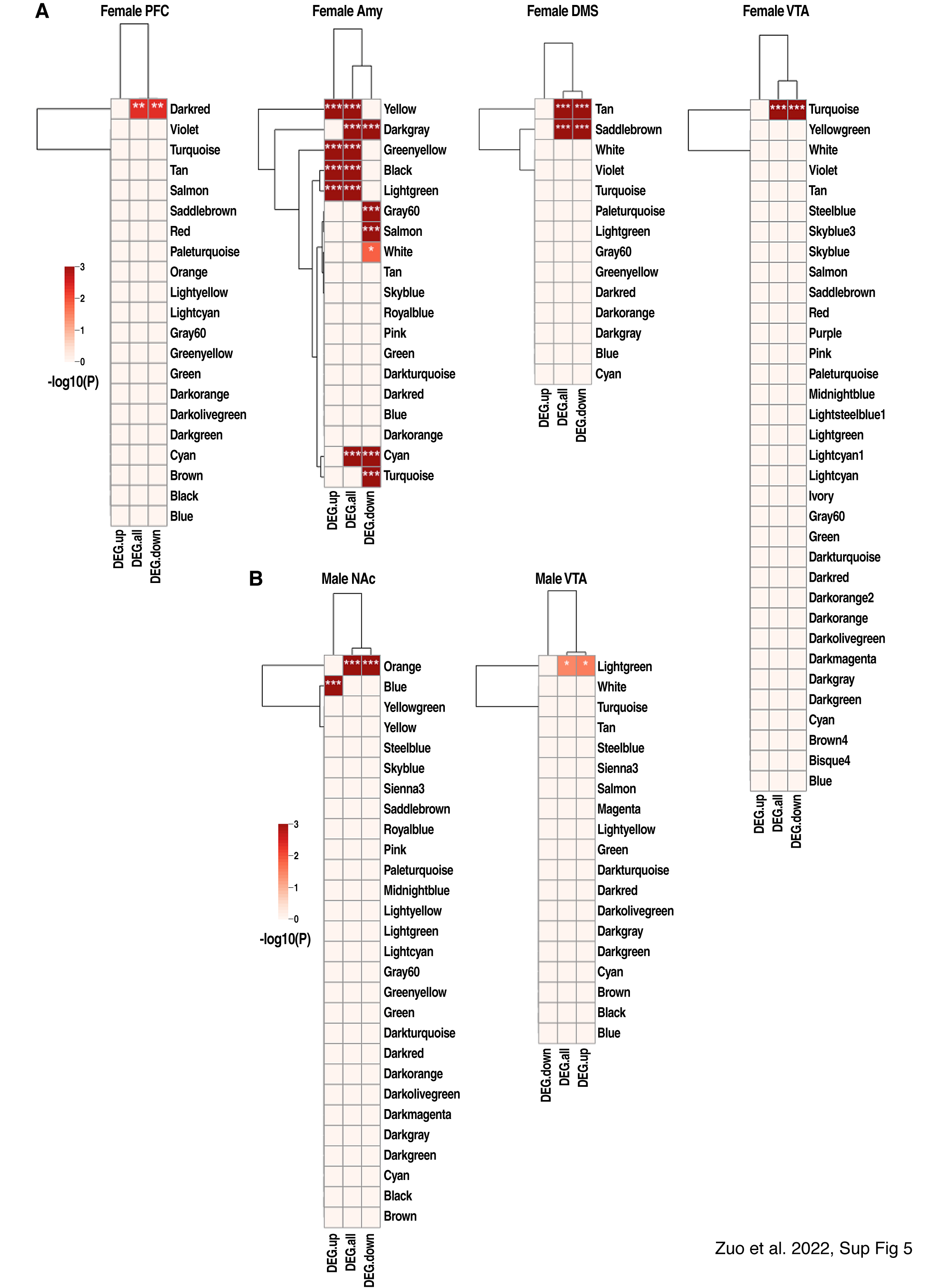

Supplement: Supplementary file 8 — Supplementary figures [file 41386_2022_1413_MOESM8_ESM.zip › Zuo et al. 2022, Sup Fig 5.tif]

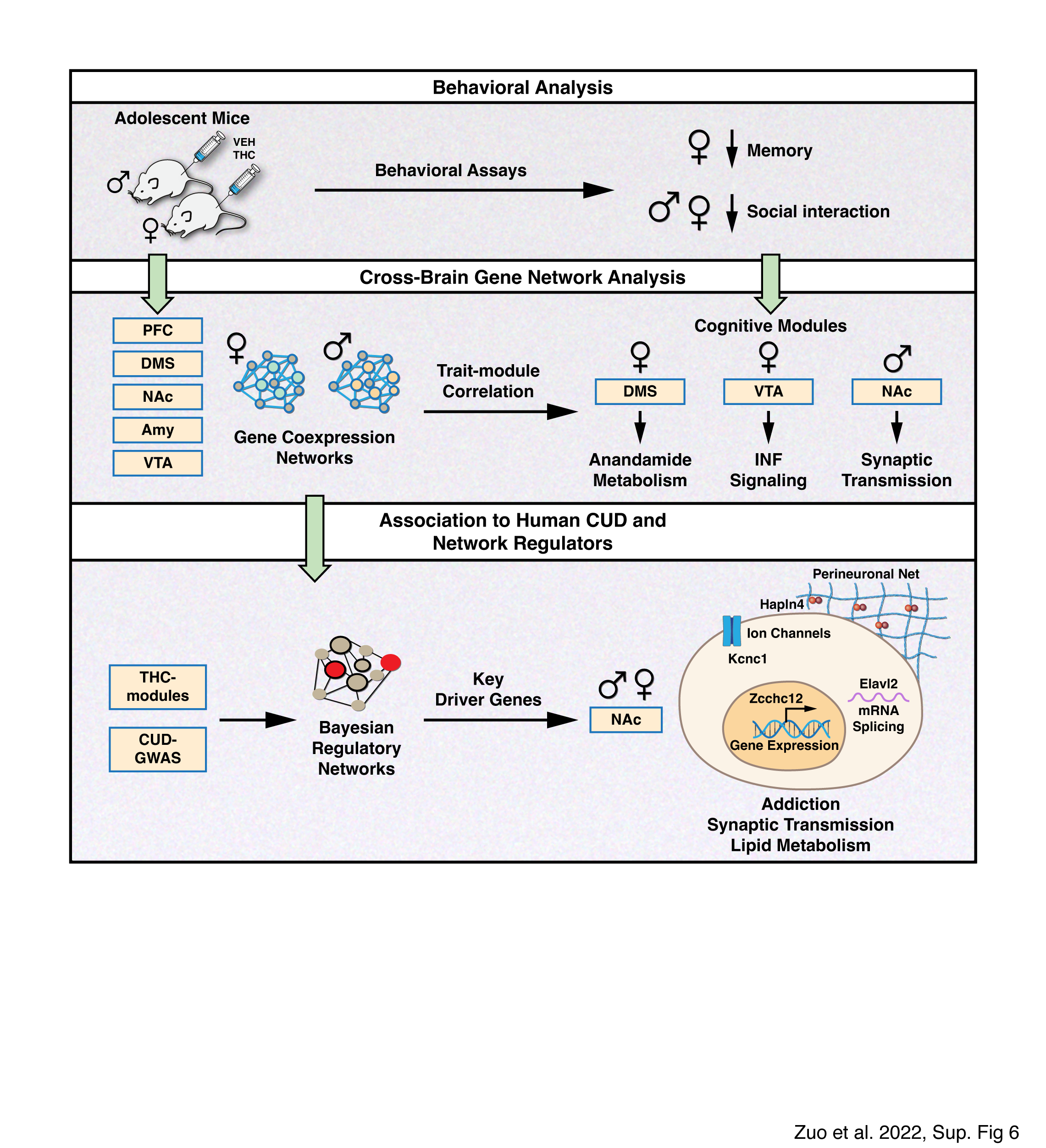

Supplement: Supplementary file 8 — Supplementary figures [file 41386_2022_1413_MOESM8_ESM.zip › Zuo et al. 2022, SUP Fig 6.tif]
